# Editorial: Advances in Plant-Hemipteran Interactions

**DOI:** 10.3389/fpls.2017.01652

**Published:** 2017-09-21

**Authors:** Jyoti Shah, Linda Walling

**Affiliations:** ^1^Department of Biological Sciences and BioDiscovery Institute, University of North Texas Denton, TX, United States; ^2^Department of Botany and Plant Sciences, University of California, Riverside Riverside, CA, United States

**Keywords:** effectors, piercing-sucking insects, resistance genes, saliva, small RNA, organismal interactions, plant defense, insect vectors

## Hemipterans

Hemipterans (e.g., aphids, whiteflies, stinkbugs, leafhoppers, and planthoppers) encompass a large group of insects with mouthparts specially modified for piercing and consuming fluids from the host (Capinera, 2008). Many hemipterans are important pests of plants and vector viral and bacterial diseases. Plant defenses against hemipterans include mechanisms that physically hinder insect feeding, as well as mechanisms that interfere with insect physiology and behavior (Painter, 1951; Kogan and Ortman, 1978; Smith, 2005). In some cases plants can alter their physiology to tolerate infestation without any detrimental effect on growth and development. Endosymbionts and phytopathogens present in the hemiptera impose an additional layer of organismal complexity to plant-hemipteran interactions. Considering the multiple organismal interactions involved, plant-hemipteran interaction studies have been conducted at different levels. This Research Topic brings together 16 manuscripts, which include a blend of reviews and research papers that address the physiology and molecular biology of plant-hemipteran interactions at these different levels.

## Plant-hemipteran interactions: what determines resistance versus susceptibility?

Host-plant resistance is a heritable trait that has been employed in breeding programs to control diseases and insect infestation. Recognition of the pest is the first step in engaging the downstream defense machinery, which in many cases involves plant hormones. In plant-pathogen interaction the involvement of *Resistance* (*R*) genes in recognition of pest-derived factors or factors produced in response to infection is well known. Several resistance (*R*) genes conferring resistance to pathogens have been cloned. However, very few *R* genes conferring resistance against hemipterans have been identified. *Vat* (virus aphid transmission), which confers resistance to cotton-melon aphid (*Aphis gossypii*) in melon, *Mi-1* which confers resistance against potato aphid (*Macrosiphum euphorbiae*) in tomato, and rice *Bph14* and *Bph26* that confer resistance to brown planthopper (*Nilaparvata lugens*) are a few that have been described. Some of these genes are unique in that they confer resistance against more than just hemipterans. For example, *Mi-1* confers resistance against potato aphid, root-knot nematode (*Meloidogyne incognita*) and whitefly (*Bemisia tabaci*) (Milligan et al., 1998; Rossi et al., 1998; Vos et al., 1998; Nombela et al., 2003) and *Vat* confers resistance against *A. gossypii*, as well as cucumber mosaic virus transmitted by *A. gossypii*, but not by other vectors (Dogimont et al., 2014). Boissot et al. review the history of the discovery of the *Vat* locus, its effect and durability against aphids, and *Vat*-conferred resistance against viruses.

A complex relationship between hormones, both cooperative as well as antagonisitic interactions, further fine-tunes defenses. However, some pests have evolved to exploit these interactions between plant hormones for their benefit to facilitate infestation. Sanchez et al. studied the relationship of hormone signaling in host specialization by pea aphid (*Acyrthosiphon pisum*, a legume specialist). They show that pea aphids perform better on their native hosts due to their ability to manipulate to their advantage the host's defense hormone pathways, in particular salicylate and jasmonate signaling.

Ji et al. have taken a genomic approach to address the question of host specialization. They compared the transcriptome of young and adult green peach aphid to that of pea aphid and found substantial changes in expression of genes involved in the metabolism and detoxification of xenobiotics between the two aphids, thus leading the authors to suggest that the ability to adapt to secondary metabolites may contribute to the host-plant adaptation by these two aphids.

Although resistance is largely viewed as the process by which plant mechanisms adversely impact pest behavior, growth, fecundity and survival, plants also have the ability to tolerate insect infestation. Unlike the classical defenses, tolerance does not adversely impact the pest. Rather, tolerance involves physiological changes in the host that alleviate the adverse impacts of herbivory on plant fitness. Koch et al. discuss the compensatory changes in plant physiology that likely contribute to tolerance, including alterations in photosynthetic rate and increase in detoxification mechanisms to counteract the damaging effects of insect infestation.

## The important contribution of saliva to plant-hemipteran interaction

Hemipteran saliva, which contains a variety of factors including proteins, is an important component of the hemipteran, which comes in direct contact with the host cells. It is intermittently released through the stylets into the host tissue. Similar to effectors released by pathogens, some salivary components have been demonstrated to facilitate infestation, while others elicit host defenses (Elzinga and Jander, 2013; Rodriguez and Bos, 2013; Kaloshian and Walling, 2016). Thus, salivary components likely contribute to the host range of the insect. van Bel and Will review what is currently known about aphid saliva, beginning with the secretion of saliva, the types of saliva, the methods of collecting saliva, and the protein components of the saliva and their likely biochemical function and impact on plant-hemipteran interaction.

In recent years, tools for transiently delivering recombinant salivary proteins have been developed for some model plants. To study the impact of aphid saliva on host selection, these tools need to be applied to different hosts. Guy et al. describe the development of an *Agrobacterium*-based tool to deliver recombinant salivary proteins to *Medicago sativa* (alfalfa) and *Pisum sativa* (pea), two important hosts of the pea aphid (*Acyrthosiphon pisum*). These tools should facilitate studying the contribution of salivary proteins on host specialization by the related aphids.

Kettles and Kaloshian utilized transient expression tools to demonstrate the effector activity of the potato aphid salivary protein Me47, which facilitates aphid infestation in tomato (*Solanum lycopersicum*) and *Nicotiana benthamiana*. However, in *Arabidopsis thaliana*, Me47 has the opposite effect in that it adversely impacts infestation, likely by eliciting host defenses. The ability of some salivary proteins to promote infestation in one host and limit infestation in others, could potentially contribute to host specialization.

## Role of small RNA and epigenomics in influencing plant-hemipteran interactions

The role of non-coding small RNA (sRNA) in regulating biological processes has become more apparent in recent years. sRNAs are involved in epigenetic regulation of gene expression, post-transcriptional control of transcript abundance as well as translational control. Although, our understanding of sRNA involvement in plant-hemipteran interaction is still in its infancy, progress made to-date has uncovered the potential involvement of several sRNAs in this interaction. Sattar and Thompson review the developments in this evolving field of sRNA in plant-hemipteran interaction. They summarize the synthesis and the potential contribution of plant-derived sRNAs to plant defense. sRNAs are found in phloem and likely consumed by the hemipterans, where they could impact processes in the insect. Hemipterans also have the machinery to synthesize sRNA that could influence insect growth and development. Further, the anti-viral RNAi machinery in the host and insect could also impact the interaction between plants, hemipteran, and their viruses.

Can hemipterans deliver sRNA into the plant? That is indeed the implications of the research paper by Van Kleeff et al. who show that whitefly sRNA can be recovered from the phloem and leaf of the host plant. Potential targets of these genes in the host have been predicted, raising the interesting possibility of the involvement of hemipteran-delivered sRNA in cross-kingdom interactions.

Finally, Kim et al. review the contribution of sRNA to epigenetic regulation of gene function in microbes with reduced genomes and its potential contribution to the regulation of genes in the aphid endosymbiont *Buchnera*. Thus, a full circle of sRNA engagement at multiple levels potentially could impact the outcome of plant-hemipteran interactions.

## Vectoring of pathogens by hemipterans

The interaction between plants, viruses and hemipteran vectors has been studied extensively in recent years and covered in recent reviews (Blanc et al., 2014; Gray et al., 2014; Gilbertson et al., 2015; Whitfield et al., 2015). In comparison, the multi-organismal interaction between plants, bacteria and hemipterans is poorly understood. Perilla-Henao and Casteel review recent progress on understanding this interaction between plants, bacteria and hemipterans, and the approaches utilized.

Phytopathogen transmission and infection is influenced by both factors in the vector and the host. Heat shock proteins (HSPs) are chaperone proteins that interact with other proteins and are involved in cellular homeostasis. HSPs also influence viral infection, which is the subject of the review by Gorovits and Czosnek, who discuss the role of HSP70 and HSP90 in plant and whitefly, respectively, on *Tomato yellow leaf curl virus* (TYLCV) life cycle and acquisition of virus by the vector. Transmission of TYLCV by whitefly is dependent on the cyclophilin CypB, the evidence for which is presented in the paper by Kanakala and Ghanim. They show that CypB interacts with TYLCV and that the transmission of TYLCV is adversely impacted when either this interaction or the activity of CypB is blocked, thus implicating an important role for CypB in transmission of TYLCV by whitefly.

## Interplay between the environment and insect infestation of plants

The environment, including water and mineral nutrient availability, temperature and presence of other organisms are some of the factors that influence host-pest interaction. Conversely, insect infestation also influences the relationship of the plant with its immediate environment, some for the better and some for the worse. Nachappa et al. studied the effect of drought on infestation of soybean plants by soybean aphid as well as the effect of drought on *Soybean mosaic virus* transmission by the soybean aphid. They report a complex effect of drought on soybean aphid population growth, viral infection, and viral transmission by the aphid. They suggest that the effect of drought on phloem amino acid content and the defense hormones SA and JA, impacts the aphid population and viral transmission.

Guo et al. studied the effect of elevated CO_2_ on the performance of whiteflies and TYLCV in tomato plants and compared the impact of the tomato *Mi1.2* gene in these two types of interactions. They observed that elevated CO_2_ did not influence insect fitness or its ability to transmit virus in the resistant (*Mi1.2*) or susceptible (*mi1.2*) genotypes. In contrast, elevated CO_2_ increased resistance to TYLCV in *mi1.2* plants, while it increased susceptibility to TYLCV in *Mi1-2* plants, thus suggesting that *Mi1.2* deployment under elevated CO_2_ conditions might increase vulnerability to TYLCV infections.

Insect infestation of the foliage has previously been shown to alter root physiology (Nalam et al., 2013). Kong et al. further report that the root microbiome is also impacted in plants experiencing a foliar whitefly infestation. The whitefly infestation-induced alteration in microbiome included enrichment of microbial species that are detrimental to whitefly, thus suggesting that root microbiome changes could potentially benefit the host plant.

## Conclusions

The range of activities being undertaken by plant-hemipteran interaction researchers to understand the physiological mechanisms and molecular factors that influence these interactions is highlighted in this Research Topic. Although still in its infancy, these studies have begun to provide insights that will have far-reaching implications at different levels, including the development of novel strategies for plant protection against hemipterans, as well as the vectored pathogens.

## Author contributions

JS and LW contributed to the drafting, editing and finalizing this editorial.

### Conflict of interest statement

The authors declare that there search was conducted in the absence of any commercial or financial relationships that could be construed as a potential conflict of interest.
